# *Akkermansia muciniphila* in neurological disorders: mechanisms and therapeutic potential via the gut-brain axis

**DOI:** 10.3389/fnins.2025.1650807

**Published:** 2025-09-11

**Authors:** Jingzhi Feng, Xiaomin Hu, Jiancheng Liu, Wenchun Wang, Liuyi Chen, Rizhao Pang, Anren Zhang

**Affiliations:** ^1^School of Health Preservation and Rehabilitation, Chengdu University of Traditional Chinese Medicine, Chengdu, China; ^2^Department of Rehabilitation Medicine, General Hospital of Western Theater Command, Chengdu, China; ^3^Sichuan Provincial Clinical Medical Research Center for Traditional Chinese Medicine Orthopedics and Sports Rehabilitation, Chengdu, China; ^4^School of Acupuncture and Tuina, Chengdu University of Traditional Chinese Medicine, Chengdu, China; ^5^Department of Rehabilitation Medicine, Shanghai Fourth People's Hospital Affiliated to Tongji University, Shanghai, China

**Keywords:** neurological disorders, *Akkermansia muciniphila*, gut microbiota, gut-brain axis, intestinal barrier

## Abstract

In recent years, the role of *Akkermansia muciniphila (A. muciniphila)* in neurological diseases has attracted increasing attention. As a probiotic, *A. muciniphila* is closely associated with host health, metabolism, and immunity, demonstrating therapeutic potential in various conditions such as obesity, atherosclerosis, inflammatory bowel disease, diabetes, and liver disorders. In the context of neurological diseases, *A. muciniphila* significantly influences the host brain through the microbiota–gut–brain axis (MGBA). This review summarizes the roles and mechanisms of *A. muciniphila* and its active components (e.g., the outer membrane protein *Amuc_1100*, extracellular vesicles *AmEVs*, and short-chain fatty acids *SCFAs*) in various neurological disorders, including Alzheimer’s disease (AD), Parkinson’s disease (PD), depression, cerebral palsy (CP), epilepsy (EP), autism spectrum disorder (ASD), and amyotrophic lateral sclerosis (ALS). It exerts protective effects by enhancing the intestinal barrier, regulating lipid metabolism, producing *SCFAs*, secreting neuroactive substances, and inhibiting neuroinflammation, thereby suggesting novel therapeutic avenues for neurological disorders. However, due to limited data from large-scale human clinical trials and the complexity of disease mechanisms and host–microbiota interactions, its clinical translation faces considerable challenges. Future efforts should focus on multicenter randomized controlled trials and in-depth mechanistic studies utilizing technologies such as metabolomics to facilitate evidence-based clinical application.

## 1 Introduction

**Figure 1:**
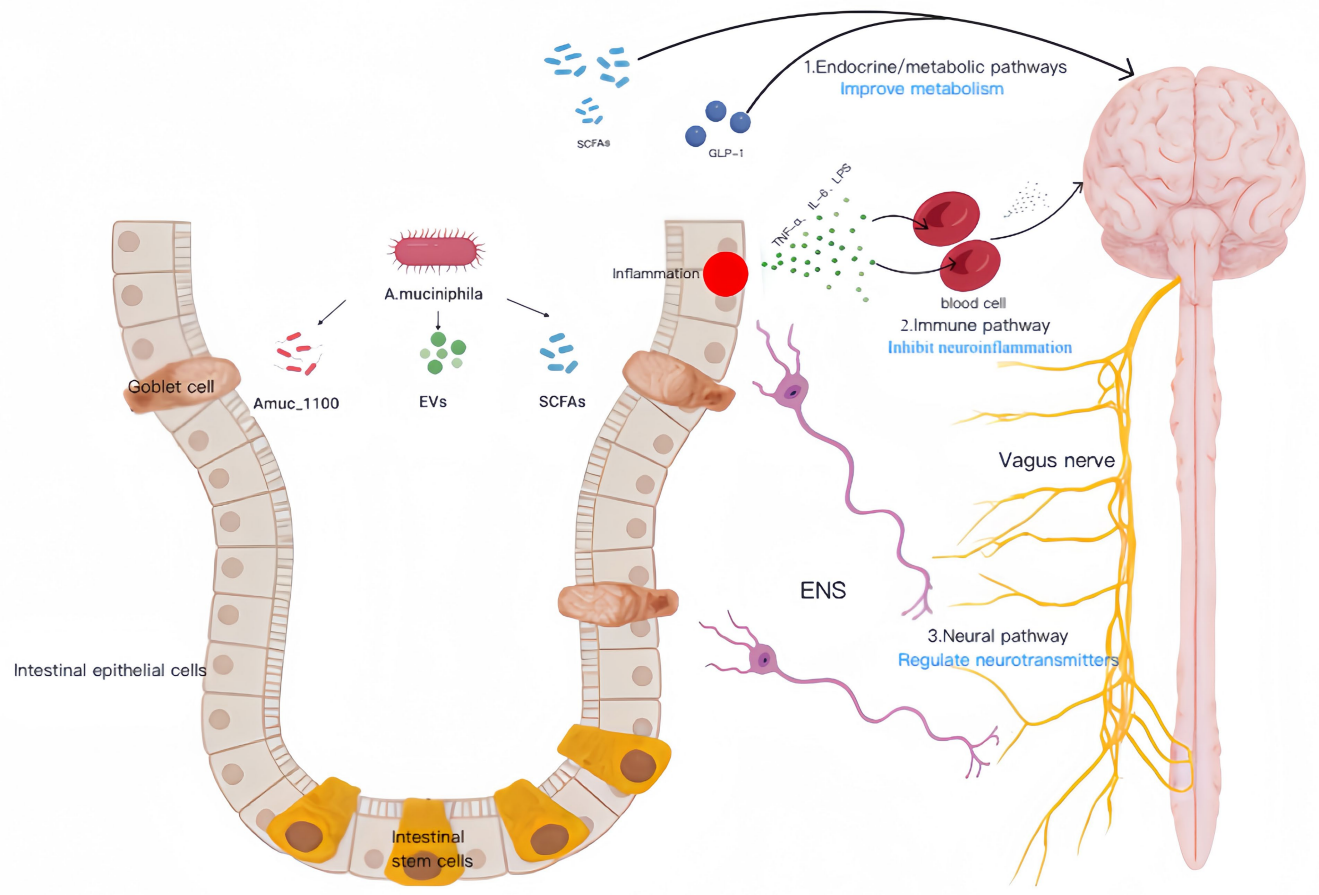
This picture explains the mechanism by which *A. muciniphila* acts on intestinal epithelial cells through its protein *Amuc_1100*, extracellular vesicle *EVs* and metabolite *SCFAs*, especially stimulating goblet cells to enhance mucus barrier function, and influencing intestinal stem cells to promote epithelial repair and renewal, jointly maintaining the health of the intestinal barrier. And it may affect brain function through the gut-brain axis.

Neurological diseases (NDs) represent a major global health challenge. According to the Global Burden of Disease Study 2016, neurological disorders (excluding infectious neurological diseases, stroke, and cancers of the brain or nervous system) ranked as the second leading cause of death worldwide (GBD 2021 Nervous System Disorders Collaborators, 2024), affecting approximately one-sixth of the global population. With ongoing population growth and aging, the associated economic burden is projected to increase further (Zhou et al., 2021). In recent years, the role of the microbiota–gut–brain axis in NDs has gained significant attention. Research conceptualizing the central nervous system, autonomic nervous system (including the enteric nervous system), digestive tract, and gut microbiota as an integrated entity has led to the establishment of the “microbiota–gut–brain axis” framework, revealing close connections between gut microbiota and nervous system function (Macpherson et al., 2023). As a next-generation probiotic, *A. muciniphila* has attracted considerable interest due to its broad health-promoting effects. It demonstrates substantial potential not only in intestinal health but also in modulating the nervous system, exhibiting beneficial effects in various NDs and thereby offering new perspectives for developing novel treatment strategies (Figure 1). It is noteworthy, however, that although *A. muciniphila* often shows reduced abundance and protective roles in most NDs, reported changes in its abundance are sometimes inconsistent, with certain studies documenting increases (as summarized in Table 1).

**Table 1:** Contradictory findings on *A. muciniphila* abundance changes in neurological disorders.

Experimental subjects	Detection techniques	Increased gut microbiota	Decreased gut microbiota	Researchers
223 PD patients and 137 healthy controls	16S rRNA	*Akkermansia* *Firmicutes*	*Prevotella* *Faecalibacterium* *Roseburia*	Nishiwaki et al. (2020)
31 PD patients and 28 healthy controls	16S rRNA	*Akkermansia* *Lactobacillus Bifidobacterium* *Streptococcus*	*Prevotella* *Faecalibacterium* *Roseburia* *Blautia*	Bedarf et al. (2017a)
197 PD patients and 130 healthy controls	16S rRNA	*Akkermansia* *Enterococcus* *Lactobacillus* *Shigell*	*Prevotella Faecalibacterium* *Roseburia* *Blautia*	Hill-Burns et al. (2017)
147 PD patients and 162 healthy controls	16S rRNA	*Akkermansia* *Enterobacteriac* *Lactobacillus* *Christensenella*	*Prevotella* *Faecalibacterium* *Roseburia* *Lachnospiraceae* *Zurichia*	Baldini et al. (2020)
197 PD patients and 103 healthy controls	16S rRNA	*Akkermansia* *Bifidobacterium* *Collinsella* *Christensenella* *Bilophila*	*Prevotella* *Faecalibacterium* *Roseburia* *Lachnospiraceae*	Cirstea et al. (2020)
27 PD patients and 44 healthy controls	16S rRNA	*Akkermansia* *Flavonifractor* *Bifidobacterium* *Parabacteroides*	*Prevotella* *Faecalibacterium* *Roseburia* *Lachnospiraceae*	Zapała et al. (2021)
C57BL/6 mice	16S rRNA	*Akkermansia*	*Lactobacillus*	Dodiya et al. (2020)
64 PD patients and 51 healthy controls	16S rRNA	*Akkermansia* *Bifidobacterium* *Streptococcus* *Escherichia*	*Lachnospiraceae* *Roseburia* *Blautia* *Bacteroides* *Butyricicoccus*	Vascellari et al. (2020)
71 MS patients and 71 healthy controls	16S rRNA	*Akkermansia* *Acinetobacter*	*Parabacteroides*	Cekanaviciute et al. (2017)
MCAO mice	16S rRNA	*Akkermansia* *Parabacteroides* *Anaerotruncus* *Alistipes* *Roseburia*	*Bacteroidetes*	Stanley et al. (2018)

## 2 Characteristics and distribution of *Akkermansia muciniphila*

*A. muciniphila* is an oval, Gram-negative, anaerobic bacterium and a key member of the human gut microbiota, belonging to the phylum *Verrucomicrobiota*. As the only representative of this phylum commonly found in the human intestine, it was first isolated and identified from human fecal samples in 2004 by Derrien et al. using strict anaerobic techniques at Wageningen University, the Netherlands (Derrien et al., 2004). Notably, *A. muciniphila* exhibits considerable oxygen tolerance, maintaining viability rates exceeding 1% even after 24 h of exposure to air, which has led to its reclassification as an aerotolerant anaerobe (Pellegrino et al., 2023). Furthermore, under microaerobic conditions, its gas production and growth rate increase, providing a competitive advantage over strict anaerobes in the gut environment (Ouwerkerk et al., 2016). Its optimal growth temperature is 37 °C, and its optimal pH is 6.5 (Derrien et al., 2004). *A. muciniphila* demonstrates broad host adaptability and is widely distributed in the intestines of animals and humans. It constitutes approximately 1–3% of the total gut microbiota, colonizes abundantly within the host intestinal mucus layer, and is most prevalent in the cecum (Collado et al., 2007). A*. muciniphila* exhibits strong adhesive and colonizing capabilities in the gut. It utilizes mucin as its sole carbon and nitrogen source, possessing a unique ability to degrade mucin, which allows it to occupy a specialized niche in the intestinal ecosystem. This trait also becomes a competitive advantage for *A. muciniphila* during host states of malnutrition or fasting, as demonstrated in hamster experiments showing significant proliferation in its abundance following fasting periods (Sonoyama et al., 2010).

Colonization by *A. muciniphila* occurs rapidly in early human life, reaching abundances comparable to those in healthy adults within the first year after birth (Collado et al., 2007). As research on *A. muciniphila* advances, its presence has been detected in the human oronasopharynx, biliary system, and breast milk (Geerlings et al., 2018). Breast milk can serve as a vector, transferring *A. muciniphila* from mother to infant, which explains its presence in the neonatal gastrointestinal tract (Ribo et al., 2021). Studies indicate that glycoside hydrolases secreted by *A. muciniphila* can degrade human milk oligosaccharides, facilitating its survival and colonization in the infant gut and contributing to immune system maturation (Kostopoulos et al., 2020). The colonization status of *A. muciniphila* varies significantly across age groups, showing a notable decrease with advancing age, particularly among the elderly (Guo et al., 2020). These changes may be linked to age-related alterations in mucus quality and quantity (Ioannou et al., 2025). The observed higher abundance of *A. muciniphila* in long-lived individuals (Wang et al., 2015) has intrigued researchers, suggesting its potential as an indicator of host health and aging status, as well as a biomarker of human longevity (Liu et al., 2021).

## 3 Association between *Akkermansia muciniphila* and neurological disorders

In the 1840s, experiments by William Beaumont first demonstrated that emotions can influence digestive rate, establishing the brain’s regulatory role over gut function and providing initial evidence for the existence of the gut–brain axis (Xu and Lu, 2025). The brain and gut engage in a complex bidirectional communication network: on one hand, gut microbiota and their metabolites can directly or indirectly regulate central nervous system function via neural, immune, and endocrine pathways; dysbiosis may mediate abnormal immune activation and disrupt host homeostasis, thereby contributing to the pathogenesis and progression of neurological diseases. On the other hand, the central nervous system inversely regulates gut motility, secretion, and barrier function through pathways such as the sympathetic–vagal nerves and the hypothalamic–pituitary–adrenal (HPA) axis, significantly influencing microbiota structure and abundance, thus forming a feedback loop (Mayer et al., 2022). Research shows that activation of hypothalamic arcuate nucleus POMC neurons or central leptin injection can rapidly reshape the intestinal neuro-immune microenvironment via the sympathetic nervous system and alter microbiota composition across multiple intestinal segments within hours (Toledo et al., 2025); chronic stress activates the HPA axis, promotes glucocorticoid release, directly inhibits intestinal epithelial mucin (e.g., MUC2) expression, compromises mucus layer integrity, increases intestinal permeability, and leads to reduced microbial diversity and richness (Foster et al., 2017).

Within this framework of gut–brain interactions, changes in *A. muciniphila* abundance are significantly correlated with neurological diseases. In the APP/PS1 Alzheimer’s disease (AD) model, its abundance decreases in an age-dependent manner (Harach et al., 2017); supplementation with *A. muciniphila* improves spatial learning and memory deficits in AD mice and delays the progression of brain pathology (Ou et al., 2020). Similar reductions are observed in Parkinson’s disease (PD) mouse models (Qiao et al., 2023) and in fecal microbiota analyses of children with neurological diseases such as cerebral palsy, epilepsy (Huang et al., 2019), and autism spectrum disorder (Ahrens et al., 2024; Wang et al., 2011). Based on this evidence, supplementing with *A. muciniphila* is considered a potential non-pharmacological intervention strategy, likely alleviating the pathological processes of neurological diseases through immune and metabolic regulation within the gut–brain axis.

## 4 Common mechanisms of *Akkermansia muciniphila* in neurological diseases

As a key regulator of the microbiota–gut–brain axis, *A. muciniphila* plays a significant role in neurological diseases through multi-pathway mechanisms, demonstrating considerable potential as a next-generation probiotic and therapeutic target. Its core mechanisms include repairing intestinal barrier function, improving metabolic homeostasis, producing bioactive molecules (e.g., *SCFAs*), regulating neurotransmitters and neurotrophic factors, and inhibiting neuroinflammation. *A. muciniphila* primarily modulates the MGBA through the following pathways (Qu et al., 2024): (1) Neural Pathway: Facilitates bidirectional gut–brain communication via the vagus nerve and enteric nervous system. Dysbiosis can lead to aberrant neural signaling, impairing synaptic plasticity and cognitive function, for instance, by downregulating brain-derived neurotrophic factor (*BDNF*) expression. (2) Immune Pathway: Gut dysbiosis increases intestinal permeability, promoting the translocation of lipopolysaccharide (LPS) and pro-inflammatory cytokines (e.g., TNF-α, IL-1β) into the circulation, disrupting blood–brain barrier integrity, and inducing neuroinflammation, thereby driving Aβ deposition, Tau hyperphosphorylation, and neuronal apoptosis. (3) Metabolic Pathway: Microbial metabolites (e.g., *SCFAs* and neurotransmitters) directly participate in brain function regulation.

In summary, *A. muciniphila* exerts a key regulatory influence on the onset and progression of neurological diseases through these multifaceted mechanisms, providing a theoretical foundation and novel intervention strategies for clinical translation.

### 4.1 Restoration of intestinal barrier function

The intestinal barrier constitutes a multi-level defense system comprising mechanical, chemical, immune, and biological barriers that operate synergistically to prevent pathogens and toxins from invading the body through the intestinal mucosa (Nie et al., 2024). Among these, the mucus layer secreted by goblet cells forms a crucial chemical and immune barrier, not only physically obstructing pathogens but also participating in antigen presentation and microbial pattern recognition, thereby initiating immune defense (McGuckin and Hasnain, 2017). The association between intestinal barrier dysfunction and central nervous system diseases has attracted growing attention. Studies indicate that PD patients frequently experience early gastrointestinal symptoms (Sun and Shen, 2018), and pathological α-synuclein (α-Syn) aggregates are present in both the enteric and central nervous systems, suggesting a potential gut origin with subsequent propagation to the brain (Challis et al., 2020). AD patients exhibit gut dysbiosis, potentially related to cerebral Aβ amyloidosis (Manfredi et al., 2025); animal experiments further confirm that antibiotic-induced manipulation of the microbiota can influence neuroinflammation and amyloid pathology in AD model mice (Seo and Holtzman, 2024). Post-mortem tissue analyses from autism (ASD) and schizophrenia patients reveal downregulated expression of tight junction proteins (e.g., ZO-1, occludin) (Fiorentino et al., 2016). Additionally, the intestinal mucus layer is significantly thinner in ASD children and their first-degree relatives compared to healthy controls (Wang et al., 2011), providing direct evidence for the link between intestinal barrier impairment and neurological diseases.

*A. muciniphila* plays multiple beneficial roles in restoring intestinal barrier function. This bacterium can significantly increase goblet cell density (Kim et al., 2021; Rodriguez et al., 2015; Yu et al., 2022) and induce a nearly three-fold thickening of the colonic mucus layer (van der Lugt et al., 2019). The underlying mechanisms primarily include: (1) Metabolizing mucin proteins to generate short-chain fatty acids (*SCFAs*), which provide energy for intestinal epithelial cells and promote mucin synthesis. For instance, in a zebrafish model of T2DM with depression, pasteurized *A. muciniphila* restored barrier integrity and reduced inflammatory factor translocation by enriching SCFA-producing *Cetobacterium* and inhibiting pro-inflammatory *Aerococcus* (Qu et al., 2025). (2) Mucin degradation products activate compensatory secretion by goblet cells, establishing a “mucus secretion—*A. muciniphila* colonization” positive feedback loop that maintains dynamic renewal of the mucus layer (Zhao et al., 2024). *In vitro* experiments demonstrate that *A. muciniphila* can adhere to human colon cancer cells Caco-2 and HT-29, upregulate mRNA expression of tight junction proteins (ZO-1, occludin, claudin), and increase transepithelial electrical resistance (TEER) values (Reunanen et al., 2015; Urban et al., 2020). *In vivo* studies show that supplementing with *A. muciniphila* promotes intestinal stem cell proliferation, increases the numbers of goblet and Paneth cells, and enhances epithelial regeneration capacity (Kim et al., 2021; Qu et al., 2021). Its barrier-repairing function also involves activation of the AMPK signaling pathway and inhibition of the TLR2-mediated NF-κB pathway, thereby coordinating mucosal immune homeostasis (Shi et al., 2022). Notably, active components of *A. muciniphila*, such as the outer membrane protein *Amuc_1100* and outer membrane vesicles (*AmEVs*), retain biological activity even after pasteurization (Gu et al., 2021). *Amuc_1100* can specifically bind TLR2 and synergistically activate TLR2/TLR4 signaling, promoting anti-inflammatory IL-10 secretion and enhancing barrier function (Ottman et al., 2017; Ottman et al., 2017; Plovier et al., 2017). *AmEVs*, serving as nanovesicles carrying bioactive molecules, increase tight junction protein expression via an AMPK-dependent pathway in Caco-2 cell models and high-fat diet (HFD)-induced diabetic mice, significantly improving intestinal barrier integrity (Chelakkot et al., 2018).

### 4.2 Improvement of lipid metabolism

Metabolic syndrome, particularly characterized by insulin resistance and obesity, is a significant risk factor for cognitive dysfunction and dementia (Bruce-Keller et al., 2009; Kordestani-Moghadam et al., 2020). Obesity can trigger the secretion of pro-inflammatory cytokines from adipose tissue, leading to systemic low-grade inflammation; this inflammatory state further induces synaptic dysfunction through pathways such as IL-1 signaling, accelerating neurodegeneration and memory decline (Erion et al., 2014). Long-term high-fat/high-sugar diets reduce gut microbiota diversity, with the depletion of *A. muciniphila* being especially pronounced (Everard et al., 2013; Schneeberger et al., 2015; Wu et al., 2020; Yang et al., 2019). Clinical studies consistently show markedly lower abundance of this bacterium in obese individuals: a qPCR-based analysis (*n* = 32) indicated significant enrichment of *A. muciniphila* in normal-weight individuals (BMI 19–24.99 kg/m^2^), while it was significantly reduced in the obese group (BMI > 30 kg/m^2^) (Teixeira et al., 2013). This phenomenon is also observed in obese pregnant women (Santacruz et al., 2010) and overweight preschool children (Karlsson et al., 2012). In animal experiments, an 8-week HFD intervention reduced *A. muciniphila* abundance by two orders of magnitude (10^9^ → 10^7^ CFU/g feces) (Everard et al., 2013).

Supplementation with *A. muciniphila* can effectively ameliorate lipid metabolism disorders and cognitive function. In diet-induced obesity or non-alcoholic steatohepatitis (NASH) models, this bacterium can reverse insulin resistance, metabolic endotoxemia, body fat accumulation, and abnormal brain glucose metabolism (Higarza et al., 2021; Mruk-Mazurkiewicz et al., 2024), through mechanisms that include improving peripheral free fatty acid and glucose metabolic utilization and enhancing insulin sensitivity (Ou et al., 2020). Furthermore, it can alleviate neuroinflammation (e.g., microglial infiltration), promote hippocampal neurogenesis and synaptic plasticity, thereby enhancing learning and memory performance (Yang et al., 2019). Certain subspecies (e.g., *A. muciniphila* sub) can also regulate tryptophan metabolism, promote hippocampal Nissl body accumulation, and further improve spatial memory (Wu et al., 2020). Notably, the protective effect of *A. muciniphila* on cognitive function is partially achieved indirectly through the improvement of lipid metabolism. In a zebrafish model of comorbid diabetes and AD (TA), pasteurized *A. muciniphila* reduced fasting blood glucose, BMI, and triglycerides, while increasing high-density lipoprotein (HDL-C); the latter promotes Aβ clearance via the blood–brain barrier. Concurrently, the bacterium stimulates intestinal L cells to secrete GLP-1, which not only improves peripheral insulin sensitivity and lipid metabolism but also enhances hippocampal function by activating central insulin signaling, forming a “peripheral–central” positive regulatory loop (Qu et al., 2023). It also remodels microbiota structure and confers metabolic benefits by modulating immune responses (Higarza et al., 2021); different strains may elicit differential immune responses (Wu et al., 2020). Human clinical studies further corroborate its metabolic regulatory effects. A trial involving 40 overweight/obese subjects with insulin resistance found that oral administration of either live or pasteurized *A. muciniphila* for 3 months significantly improved weight, insulin sensitivity, and other metabolic indicators (Mruk-Mazurkiewicz et al., 2024), with inactivated preparations (containing *AmEVs*) potentially offering superior efficacy (Ashrafian et al., 2019), thereby providing a metabolic basis for intervening in cognitive impairment.

### 4.3 Short-chain fatty acids

*A. muciniphila* synthesizes short-chain fatty acids (SCFAs), including acetate, propionate, and butyrate, primarily through the breakdown of gastrointestinal mucin (Xia et al., 2022). These metabolites serve not only as crucial energy sources for colonocytes and gut microbes but also repair intestinal epithelial damage and enhance physical barrier function by upregulating tight junction protein expression (e.g., occludin, ZO-1), thereby inhibiting pathogen and toxin translocation (Li X. et al., 2022). *SCFAs* can cross the blood–brain barrier and may also indirectly influence central nervous activity via the enteric nervous system and vagus nerve. Functioning as extracellular signaling molecules, *SCFAs* primarily trigger downstream responses by binding to G-protein-coupled receptors (GPCRs); for example, butyrate and propionate can promote the secretion of gut hormones like glucagon-like peptide-1 (GLP-1) and peptide YY (PYY), which are involved in energy balance and glucose regulation (Cunningham et al., 2021; Jiao et al., 2021).

*SCFAs* demonstrate significant regulatory potential in neurological diseases. In Parkinson’s disease (PD), patients often exhibit decreased fecal SCFA levels alongside increased plasma SCFA levels, an abnormal pattern significantly correlated with the degree of specific microbiota dysbiosis and disease severity (Yang et al., 2022); supplementing with *A. muciniphila* or its derived *SCFAs* can alleviate neuroinflammation, promote neurogenesis, and improve motor function and dopaminergic neuron survival in PD mice (Hou et al., 2021; Qiao et al., 2024). Furthermore, *SCFAs* also play regulatory roles in neurodevelopmental and psychiatric disorders. Reduced SCFA levels are commonly reported in depression and anxiety models (Li Z. et al., 2022); oral sodium propionate can ameliorate depressive-like behaviors by increasing central histone H3 acetylation levels and activating *BDNF* expression, among other mechanisms (Behrens et al., 2024). Clinical studies also show an inverse correlation between circulating SCFA levels and depression severity, with higher baseline levels predicting a better treatment response (Schiweck et al., 2025). High concentrations of butyrate can enhance histone acetylation, improving social behavior and cognitive flexibility in autism (ASD) model mice (Kratsman et al., 2016); propionate may influence ASD pathogenesis by regulating neurotransmitters, inflammation, and mitochondrial function (Anaclerio et al., 2024). In schizophrenia models, SCFA supplementation alleviates social deficits and sensory gating abnormalities (Dalile et al., 2019), and a clinical trial demonstrated that butyrate improved cognitive function in first-episode patients (Li et al., 2021).

In summary, *SCFAs*, serving as key effector molecules through which *A. muciniphila* exerts its functions, not only maintain intestinal barrier and metabolic homeostasis but also regulate central nervous system function through immune, epigenetic, and neuroendocrine pathways, presenting potential targets for therapeutic intervention in various neuropsychiatric disorders.

### 4.4 Neuromodulatory substances

Gut microbiota can modulate the levels of neurotransmitters and influence the expression of key synaptic plasticity proteins such as NMDA receptors and brain-derived neurotrophic factor (*BDNF*), thereby dynamically regulating neural function (Colucci-D’Amato et al., 2020). Among these, *BDNF* is closely associated with neuronal regeneration and repair, and its levels are significantly reduced in the brains of depression patients (Cavaleri et al., 2023). Serotonin (5-HT), an important monoamine neurotransmitter and a critical gut–brain axis signaling molecule involved in neural plasticity regulation, is also found at lower concentrations in depression patients (Cui et al., 2024). *A. muciniphila* plays a key role in regulating mood and preventing depression. In a chronic restraint stress-induced depression model, *A. muciniphila* restored hypothalamic–pituitary–adrenal (HPA) axis function, regulated corticosterone levels, reestablished dopaminergic signaling homeostasis, and restored hippocampal *BDNF* expression (Ding et al., 2021). In an alcohol exposure combined with chronic stress model, *A. muciniphila* exerted antidepressant effects by promoting intestinal 5-HT levels, reducing serotonin transporter (SERT) expression, and inhibiting cFos activation in the enteric nervous system, thereby altering gut-to-brain signaling (Guo et al., 2024). *A. muciniphila* and its outer membrane protein *Amuc_1100* can alleviate antibiotic-induced anxiety and depression by modulating the *BDNF*/TrkB signaling pathway or increasing serum and hippocampal 5-HT levels (Sun et al., 2023). Observations of decreased *A. muciniphila* abundance in ALS patients were further corroborated by a close association with reduced blood and brain levels of nicotinamide (NAM); supplementing with NAM effectively alleviated clinical symptoms in ALS mice (Blacher et al., 2019). Animal model studies indicate that colonizing with *A. muciniphila* can increase central nervous system nicotinamide levels by regulating the tryptophan–nicotinamide metabolism pathway, thereby improving mitochondrial and motor neuron function and alleviating motor dysfunction in ALS mice (Blacher et al., 2019). However, the precise mechanism by which *A. muciniphila* promotes nicotinamide production remains to be fully elucidated.

### 4.5 Suppression of neuroinflammation

Bacterial-derived lipopolysaccharide (LPS) and amyloid proteins can induce abnormal increases in intestinal permeability, promoting the overexpression of pro-inflammatory factors. These pro-inflammatory factors can migrate to the central nervous system (CNS) through compromised intestinal and blood–brain barriers (BBB), triggering brain immune responses (Acioglu and Elkabes, 2025). Significantly elevated levels of pro-inflammatory cytokines (e.g., IL-1, IL-6, TNF-α, TGF-β) play a pivotal role in neuroinflammation (Bagyinszky et al., 2017; Wang et al., 2018). Persistent neuroinflammation has been established as a factor accelerating the progression of certain neurodegenerative diseases (Zhang et al., 2023). Studies indicate that *A. muciniphila* can mitigate chronic low-grade inflammation through various mechanisms: including upregulating concentrations of fat-soluble anti-inflammatory factors (e.g., α-tocopherol and β-sitosterol), inhibiting JNK phosphorylation, and increasing IKBα expression, thereby blocking downstream signaling of LPS/Binding Protein (LBP) (Zhao et al., 2017). Although live bacteria are often considered the primary form for probiotic effects, pasteurized *A. muciniphila* and its main outer membrane protein *Amuc_1100* can produce comparable or even stronger anti-inflammatory effects. They significantly inhibit macrophage infiltration and cytotoxic T lymphocyte activation (Wang et al., 2020), reduce systemic inflammation, and consequently improve glycemic control and spatial memory (Wu et al., 2020).

**Figure 2:**
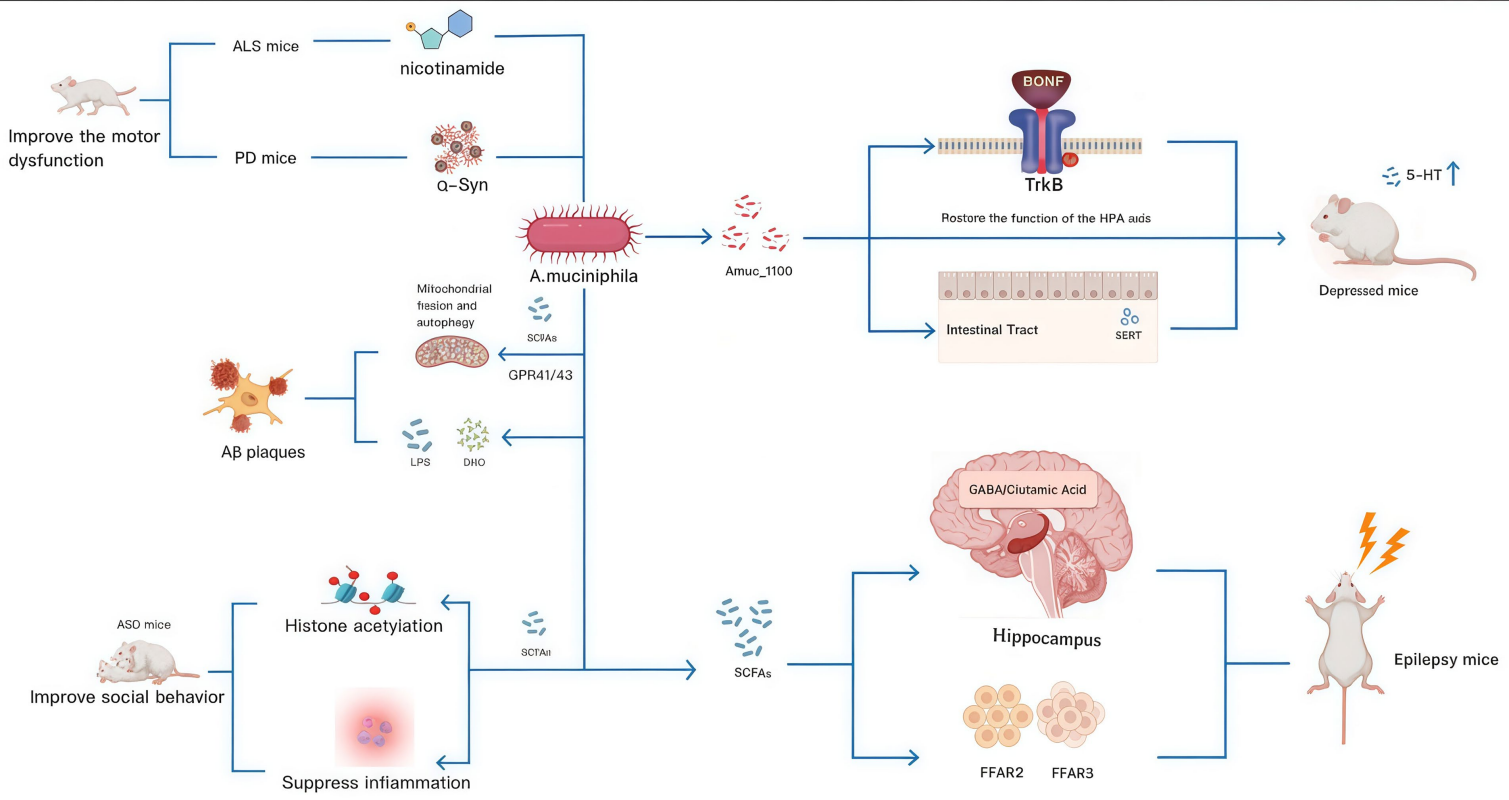
The role and specific mechanism of *A. muciniphila* in different neurological diseases.

*A. muciniphila* demonstrates potent anti-neuroinflammatory effects across various disease models. In an Alzheimer’s disease (AD) model, supplementation with *A. muciniphila* reduced serum LPS and intestinal diamine oxidase (DAO) concentrations, decreased cerebral Aβ plaque deposition, and improved spatial learning and memory performance (Ou et al., 2020). Microglial inflammation is a key process in various neuropsychiatric diseases; *A. muciniphila* treatment inhibited hippocampal microglial proliferation, restored neuronal development and synaptic plasticity, thereby reversing HFD-induced hippocampal-dependent cognitive impairment (Yang et al., 2019). In an alcohol-LPS combination-induced mouse model, this bacterium reduced serum LPS and pro-inflammatory cytokine (TNF-α, IL-1β) levels and corrected expression abnormalities of depression-related genes, indicating potential for intervening in alcohol-related mood disorders (Guo et al., 2022). In a zebrafish model of T2DM with depression, after 30 days of intervention with pasteurized *A. muciniphila*, pro-inflammatory cytokine (IL-6, TNF-α, IFN-γ) levels significantly decreased, and anti-inflammatory IL-4 expression increased, resulting in the direct inhibition of neuroinflammation (Qu et al., 2025). Further investigation revealed that its active component *Amuc_1100* achieves its antidepressant effect through multiple pathways: regulating gut microbiota composition, elevating brain-derived neurotrophic factor (*BDNF*) levels, and inhibiting neuroinflammatory pathways (Liu et al., 2022). This discovery clarifies the key molecular basis of *A. muciniphila*’s neuroprotective effects. Notably, beyond direct probiotic supplementation, combined intervention strategies targeting oxidative stress and inflammation also show promise for alleviating neurodegenerative pathology. For example, He et al. utilized nano-bubble hydrogen-rich water (HRW) intervention in an AD zebrafish model and found that HRW not only significantly reduced brain oxidative damage markers like MDA and ROS but also inhibited pro-inflammatory factors TNF-α, IL-6, IL-1β and elevated the anti-inflammatory factor IL-10. It reduced neutrophil infiltration and Aβ deposition, thereby improving neuropathology. These results complement the neuroprotective effects of *A. muciniphila*, collectively supporting the value of combined antioxidant–anti-inflammatory strategies in AD treatment (He et al., 2025) (Figure 2).

## 5 Disease-specific mechanisms

### 5.1 Alzheimer’s disease: specific mechanisms targeting aβ pathology

The core pathological features of AD are Aβ plaque deposition and tau protein tangles. Beyond alleviating AD progression by repairing the intestinal barrier (Section 3.1) and inhibiting neuroinflammation (Section 3.5), its metabolite propionate has also been shown to regulate mitochondrial fission and autophagy through GPR41/43 receptors, maintaining neuronal mitochondrial homeostasis and thereby ameliorating Aβ-induced neurotoxicity, adding another significant mechanistic dimension to the protective role of *A. muciniphila* in AD (Wang et al., 2025).

### 5.2 Parkinson’s disease: specific regulation of α-synuclein pathology

The pathological core of Parkinson’s disease is the abnormal aggregation of α-synuclein (α-Syn). A central role of *A. muciniphila* in PD involves blocking the gut–brain axis transmission of α-Syn via the vagus nerve (Sampson et al., 2016); however, clinical studies reveal contradictory observations: certain *A. muciniphila* strains may upregulate α-Syn expression (Bedarf et al., 2017b), indicating that future clinical translation necessitates personalized protocols based on strain typing.

### 5.3 Epilepsy: specific regulation of GABAergic signaling and ion channels

The core pathological feature of epilepsy is abnormal neuronal hypersynchronous discharge, associated with impaired GABAergic inhibition and voltage-gated channel dysfunction. Notably, ketogenic diets (KDs), whose effects are mediated by gut microbes, can be used to treat refractory epilepsy. 16S rRNA sequencing analysis revealed that the abundance of *A. muciniphila* in mouse intestines was significantly upregulated by KD intervention (Dooling and Costa-Mattioli, 2018; Olson et al., 2018). KD-promoted proliferation of *A. muciniphila* and *Parabacteroides* increased the hippocampal GABA/glutamate neurotransmitter ratio, effectively suppressing epileptiform discharges; these anticonvulsant effects persisted for 21 days following the microbiota intervention. Further studies found that butyrate can inhibit the epileptic process by activating G protein-coupled receptors such as FFAR2/FFAR3, which are widely expressed in the nervous system (Olson et al., 2018). Future work should explore the sequential combination of *A. muciniphila* strains with antiepileptic drugs and develop non-invasive EEG–microbiota monitoring technologies to optimize individualized treatment protocols.

## 6 Summary and outlook

Current research suggests that *A. muciniphila* may possess protective effects in various neurological diseases, but it exhibits considerable heterogeneity across different disease types, and even different stages or subtypes of the same disease (Table 1). Taking Parkinson’s disease (PD) as an example, some studies report decreased gut abundance, while others document a significant increase. This inconsistency likely stems from variations in study design, population heterogeneity (including dietary patterns, medication use, and geographical background), and functional diversity among different strains.

Although *A. muciniphila* is regarded as a highly promising target for neurological disease intervention, its clinical translation faces several safety challenges. As a Gram-negative bacterium, its outer membrane lipopolysaccharide (LPS) could potentially induce excessive inflammation or opportunistic infections in susceptible populations (e.g., immunocompromised patients) (Zhao et al., 2019), yet clinical safety data specific to these high-risk groups are currently lacking. Furthermore, its mucin-degrading and colonizing properties might exacerbate mucosal damage and increase the risk of bacterial translocation in individuals with pre-existing impairment of the intestinal barrier (e.g., those with active inflammatory bowel disease) (Derrien et al., 2017). Short-term clinical trials (≤3 months) generally report good tolerance (Depommier et al., 2019; Tang et al., 2022); however, its long-term safety (>6 months) still requires systematic evaluation, necessitating particular attention to the impact of single-strain colonization on overall gut microbiota diversity (Romaní-Pérez et al., 2021) and its effects on individual baseline microbial architectures (Zmora et al., 2018). Beyond safety concerns, other critical bottlenecks hinder its clinical application: firstly, the impact of functional heterogeneity at the strain level on efficacy remains unclear; secondly, validated individualized dosing strategies are absent. Corresponding risk mitigation strategies include: (1) Prioritizing the use of pasteurized preparations or purified active components (e.g., the outer membrane protein *Amuc_1100*, extracellular vesicles *EVs*), which significantly reduce infection risk while retaining bioactivity (Ashrafian et al., 2019; Depommier et al., 2019; Ottman et al., 2016). (2) Developing colon-targeted delivery systems to enhance local bioavailability and minimize systemic exposure. (3) Establishing treatment guidelines stratified based on immune status, avoiding use in patients with active autoimmune diseases or severe immunodeficiency. Future research should prioritize the following directions: rigorously validating long-term safety through Phase I clinical trials and strain engineering; conducting large-sample, multicenter randomized controlled trials to optimize dosing regimens (including dose, duration, and combination strategies); and integrating multi-omics approaches like metagenomics and metabolomics to systematically elucidate its mechanisms of action, ultimately paving the way for the precise application of *A. muciniphila* in the treatment of neurological diseases.
